# Supplementary material for: Toward an Early Diagnosis for Alzheimer’s Disease Based on the Perinuclear Localization of the ATM Protein
Source: Cells. 2023 Jun 29;12(13):1747. doi: 10.3390/cells12131747 (PMC10340316; doi:10.3390/cells12131747)
Supplement: Supplementary file 1 [file cells-12-01747-s001.zip › cells-2438263-supplementary.pdf]

Supplementary Data

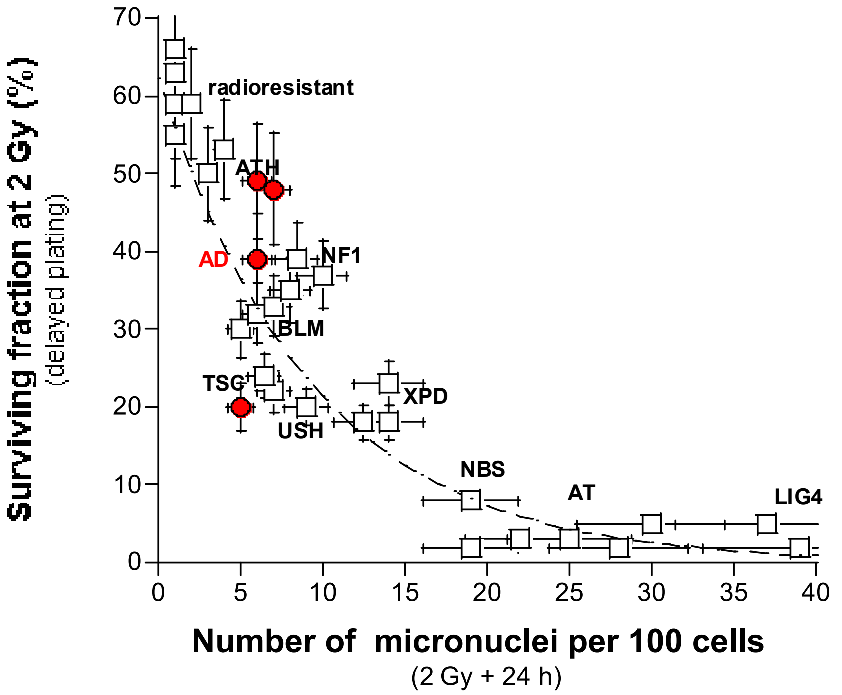

Figure S1: Correlation between SF2 and residual micronuclei data. Our data from AD cells were added to the figure 3B shown in [14]. The SF2 values (± SEM) and the corresponding numbers of micronuclei per 100 cells assessed 24 h post-irradiation (± SEM) from fibroblasts deriving from patients suffering from the indicated syndromes (open squares) (AT:ataxia telangiectasia, homozygous mutations of *ATM*; LIG4 : homozygous mutations of *LIG4*; NBS: Nijmegen's syndrome, homozygous mutations of *NBS1*; XPD, xeroderma pigmentosum D, homozygous mutations of *XPD*; USH, Usher's syndrome, homozygous mutations of USH; TSC, tuberous sclerosis , heterozygous mutation of *TSC*; Bloom's syndrome, homozygous mutations of *BLM*; NF1, neurofibromatosis type 1, heterozygous mutations of neurofibromin; ATH, heterozygous mutations of *ATM*. The red circles are the data obtained from the AD cell lines. The best data fit was obtained with the following law: y = 62.2 exp(-0.107 x); r^2=0.939 (dotted line) in quantitative agreement with [14].

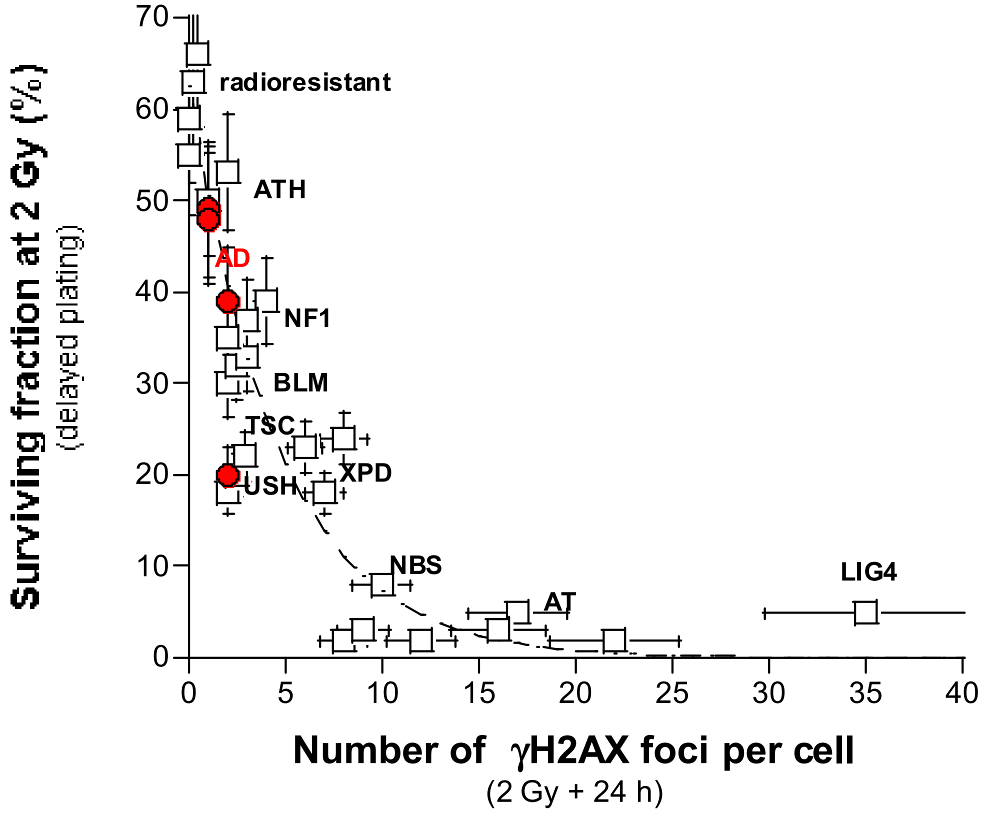

Figure S2: Correlation between SF2 and residual γH2AX foci data. Our data from AD cells were added to the figure 5B shown in [38]. The SF2 values (± SEM) and the corresponding numbers of γH2AX foci. assessed 24 h post-irradiation (± SEM) from fibroblasts deriving from patients suffering from the indicated syndromes (open squares) (AT:ataxia telangiectasia, homozygous mutations of *ATM*; LIG4 : homozygous mutations of *LIG4*; NBS: Nijmegen's syndrome, homozygous mutations of *NBS1*; XPD, xeroderma pigmentosum D, homozygous mutations of *XPD*; USH, Usher's syndrome, homozygous mutations of USH; TSC, tuberous sclerosis , heterozygous mutation of *TSC*; Bloom's syndrome, homozygous mutations of *BLM*; NF1, neurofibromatosis type 1, heterozygous mutations of neurofibromin; ATH, heterozygous mutations of *ATM*. The red circles are the data obtained from the AD cell lines. The best data fit was obtained with the law: y= 62.56 exp(-0.216 x); r^2=0.87 (dotted line). in quantitative agreement with [14].

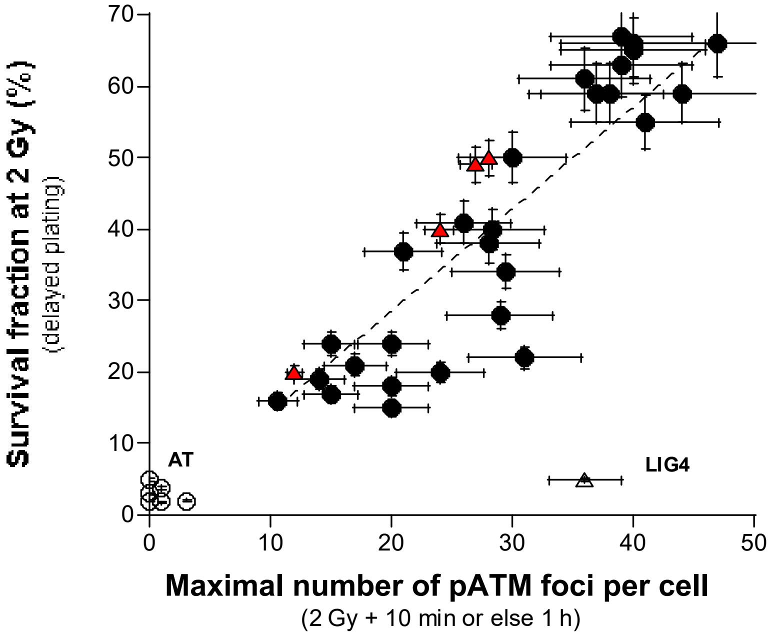

Figure S3. Correlation between SF2 and pATMmax data. Our data from AD cells were added to the figure 7A shown in [14]. The SF2 data from 36 COPERNIC cell lines were plotted against the corresponding maximal number of pATM foci per cell assessed either at 10 min or at 1h post-irradiation (closed circles). Each point corresponds to the mean ± standard error of the mean (SEM) of 3 independent triplicates, at least. The red triangles are the data obtained from the AD cell lines. The best data fit was obtained with the linear law: y=1.422 x; r^2=0.87 (dotted line).

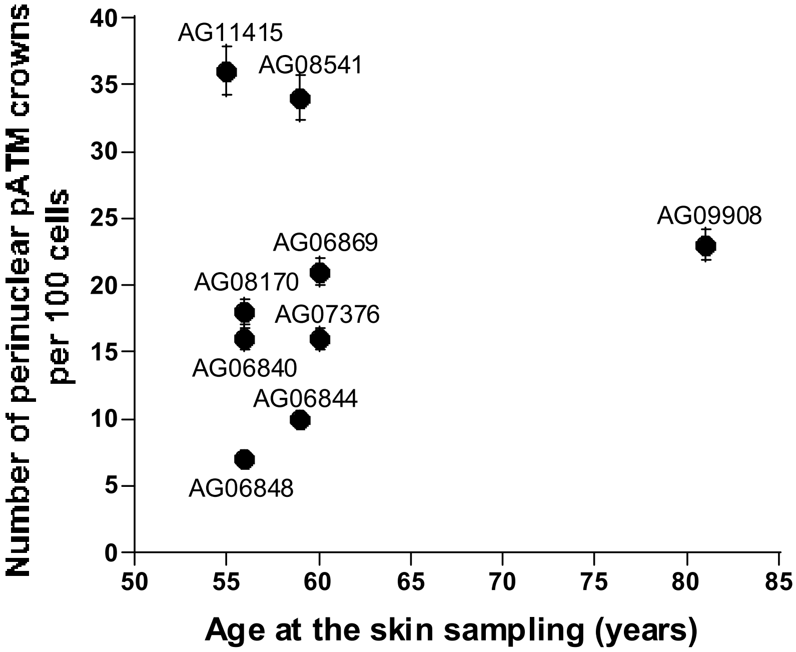

Figure S4. The number of perinuclear pATM crowns per 100 cells vs. the age at the skin sampling. The number of perinuclear pATM crowns per 100 cells shown in Fig. 3B (± SEM) were plotted against the correspondant age at the skin sampling of the donor.
